# A Forward Collision Warning System for Smartphones Using Image Processing and V2V Communication

**DOI:** 10.3390/s18082672

**Published:** 2018-08-14

**Authors:** Subhadeep Patra, Peter Veelaert, Carlos T. Calafate, Juan-Carlos Cano, Willian Zamora, Pietro Manzoni, Fabio González

**Affiliations:** 1 Department of Telecommunications and Information Processing, Ghent University, Sint-Pietersnieuwstraat 41, Ghent 9000, Belgium; 2 Department of Computer Engineering, Universidad Politécnica de Valencia, Camino de Vera S/N, Valencia 46022, Spain; calafate@disca.upv.es (C.T.C.); wilzame@posgrado.upv.es (W.Z.); 3 IPI-Imec Research Group at Ghent University, Sint-Pietersnieuwstraat 41, Ghent 9000, Belgium; Peter.Veelaert@UGent.be; 4 Faculty of Computer Science, Universidad Laica Eloy Alfaro de Manabí, Av. Circunvalación-Vía a San Mateo, Manta-Manabí 130802, Ecuador; 5 Computer Systems and Industrial Engineering Department, Universidad Nacional de Colombia, Carrera 45 #26–85, Bogotá D.C. 111611, Colombia; fagonzalezo@unal.edu.co

**Keywords:** forward collision warning, Android, smartphones, ITS, vehicular network

## Abstract

In this paper, we present a forward collision warning application for smartphones that uses license plate recognition and vehicular communication to generate warnings for notifying the drivers of the vehicle behind and the one ahead, of a probable collision when the vehicle behind does not maintain an established safe distance between itself and the vehicle ahead. The application was tested in both static and mobile scenarios, from which we confirmed the working of our application, even though its performance is affected by the hardware limitations of the smartphones.

## 1. Introduction

Intelligent Transportation System (ITS) is a technology, application or platform that, without embodying intelligence as such, aims to improve the safety, mobility and efficiency of ground transportation systems, making use of sensing, analysis, control and communications technologies. ITS includes a wide range of applications that process and share information, enabling users to be better informed, improve traffic management, minimize environmental impact and increase the benefits of transportation to commercial users and the public in general. ITS applications sometimes depend on Vehicular Networks (VNs) for communication. However, eight years after the introduction of the Institute of Electrical and Electronics Engineers (IEEE) 802.11p standard [1] for vehicular communication, vehicles used on a daily basis still lack the capability of communicating with one other. This fact impedes the use of the many ITS safety applications that take advantage of the vehicular network for data exchange. The obvious way to handle this problem is to use the available technologies at the disposal of common users to develop solutions that are easily deployable, effortless to adopt and, moreover, cost effective.

Our goal is to study the effects of integrating smartphones in VNs for the development of ITS safety applications, aiming towards rapid acceptance of these kinds of applications by the general public. The choice of smartphones here is not only justified by their wide availability and use, but also because they are evolving towards high performance terminals with multi-core microprocessors packed with a sufficiently diverse group of sensors.

Warning generation on the detection of a high probability of collision has always been a topic of interest among researchers. Early works include [2], which studied the use of radar technology for detecting possible collisions. An effort by General Motors [3] resulted in the publication of [4], dedicated to the design of a simulation tool to evaluate technical and functional specifications of Forward Collision Warning (FCW) systems based on radar sensors. A beacon-based collision warning system [5] was designed by Miller et al. in 2002 that did not require vehicles to be in the line of sight for operation.

One of the first vision-based approaches was a simple algorithm to detect vehicles and warn drivers when they are too close to the other vehicle, presented by Srinivasa [6]. An improved version [7] combining the use of data from a forward-looking camera and a radar was proposed by the same author a year later. Other approaches that rely on data from a camera for warning generation were presented in [8,9,10]. The work in [11,12] was an investigation initiated by Volvo [13] on collision avoidance and automatic braking by only making use of a car mounted with a radar and camera. Another application that used a vision-based approach, depending on Sobel edge enhancement [14] and an optical flow algorithm, is [15]. Chang et al. [16] studied the fusion of vision and Global Positioning System (GPS) sensing for collision warning.

Misener et al. [17], on the other hand, described a cooperative collision warning project that included forward collision warning, an intersection assistant, along with a blind-spot and lane change assistant. First, each vehicle estimated its position combining data from GPS with information like wheel speed, steering angle and yaw rate. Later, vehicles exchanged this information among themselves to generate warnings as required. A similar application just based on the use of GPS and motion sensors was presented in [18]. Fusion of data from Light Detection and Ranging (LiDAR) and other on-board sensors that compute vehicle speed, acceleration and brake signal was used in the solution [19] presented by Lei et al. Other related studies like [20] showed the positive effects of vibrotactile feedback on steering wheel and seat-belt for FCW systems.

Thus, we have come across sensor-based solutions that aim to generate warnings when the probability of collision is high. These solutions are mostly vision based, although there are others that rely on GPS, radar technology or LiDAR. One thing these solutions have in common is that they are dedicated systems and only applicable to scenarios for which they are designed. Our aim is to develop ITS safety applications for smartphones in order to achieve rapid acceptance by the general public. Here, we present a FCW application for smartphones, which uses vision and location data to alert not only the user of the application, but also the driver of the car in front of a probable accident. The application relies on Vehicle to Vehicle (V2V) communication for this purpose.

## 2. Application Features

The FCW application that we will present in this section is based on OpenALPR [21] and has been designed to be used on bidirectional two-lane roads in urban areas. It is aimed at Android-based smartphones or devices that possess at least a back camera and GPS. The device is to be placed on the dashboard with the camera facing ahead, and the application aims to detect vehicles ahead that are too close to the current vehicle using license plate recognition. Upon detection of the plate making use of the back camera of the Android device, we can calculate the distance of the vehicle from the one ahead since license plates have a fixed size depending on the geographical region. Note that, if more than one license plate is detected in the frame, which means there are multiple vehicles present, distance is only calculated for the vehicle ahead traveling in the same direction. Thus, we need to select only the plate of the vehicle ahead and discard the others.

**Figure 1:**
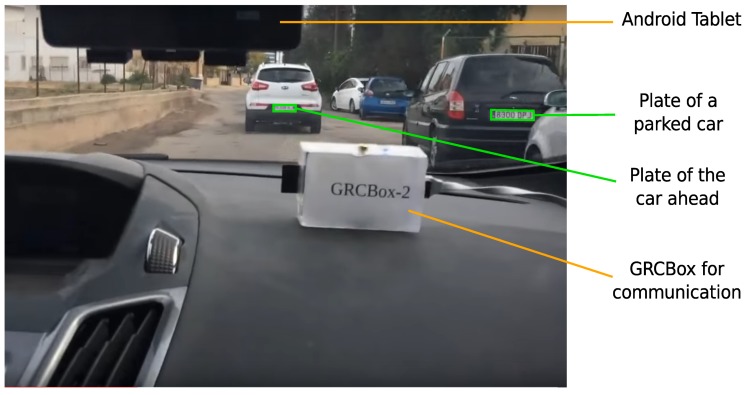
Experimental setup when testing our application.

Figure 1 shows one of the photographs taken during our experiments with our Android FCW application. It demonstrates the complete setup consisting of a dashboard-mounted Android device with GPS, and a back camera; and a GRCBox [22] device that allows inter-vehicular communication. The GRCBox is equipped with at least two wireless network interfaces: one configured to act as an access point and the other in ad-hoc mode; the device within the car with the application installed is connected to the WiFi access point provided by the GRCBox. The GRCBox forwards all data received from one interface to the other acting as a router; thus, the application is able to take advantage of the vehicular network that is created using the interface working in the ad-hoc mode. We can see from the figure that the license plate of a parked vehicle and the plate of the vehicle traveling ahead have been recognized. Cases might arise where plates of vehicles coming from the opposite direction could also be recognized. Thus, to detect and eliminate such cases, we take advantage of GPS data exchanged among cars using V2V communication.

Once cases like recognized plates of parked vehicles and those of vehicles coming from the opposite direction have been eliminated, only the license plate of the vehicle traveling ahead remains. Distance between the vehicle behind and the one ahead is calculated based on the size of the plate in the image captured. If this distance is less than the average length of a car, then alerts are generated. Furthermore, the vehicle ahead is informed of the situation using the GRCBox network.

## 3. Functional Details of the Application

The different steps that the application follows to detect situations when cars are too close to one another and generate alerts are: broadcast of GPS and other application-related data by the vehicles; recognition of the license plate of the car from the image gathered using the camera of the device in which the application is installed; selecting the plate of the vehicle ahead among all recognized plates; estimation of the distance between the vehicle ahead and the one behind; and finally, alerting the drivers of both vehicles if a safe distance is not maintained. Now, let us look at each of these steps in detail.

### 3.1. Vehicles Broadcast Message

As soon as the application is first started, the user needs to enter the vehicle plate number in which the device would be placed and used. The application checks for a location fix and GRCBox connectivity; and later, starts broadcasting messages as soon it has acquired two different location fixes, a couple of seconds apart. The two location fixes consist of the current and previous ones of the device, which are constantly updated while the application keeps running.

**Figure 2:**
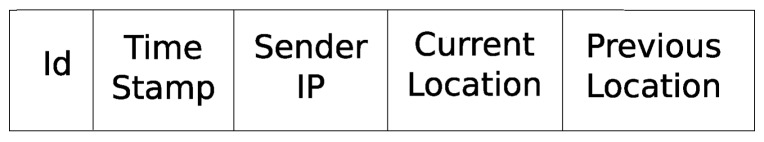
Structure of the packets used in our FCW application.

A vehicle while broadcasting data, at the same time, listens for incoming messages from other vehicles within its communication range. The messages used by our application, as can be seen in Figure 2, contain a message Id, which is the actual license plate number of the vehicle entered by the user, sender generated time stamp, the Internet Protocol (IP) address of the sender, the current and previous location of the sender.

The broadcasting of messages is only stopped when the application is closed by the user on terminating the journey. Note that only the vehicles in a one-hop radius will receive the message, as no re-broadcasting or forwarding of messages takes place. The received message is saved by the application in its database, adding a local time stamp. Entries in the database are overwritten when new packets are received from the same source and are eliminated if no update is received from the same source for a long time.

### 3.2. Plate Recognition

While the communication between devices using our application is happening in the background, at the same time, the back camera of the smartphone or tablet is used to capture images, processed to recognize license plates within the image, if any. For this purpose, the following sequence takes place: detection of possible plate regions, converting regions of interest into monochromatic images, character analysis, edge detection, deskewing, segmentation of characters, Optical Character Recognition (OCR) and the generation of results based on region-wise known templates. Let us at a look at each of these steps:

- Detect regions of interest: The first step, which is also the most processing-intensive step, involves the detection of regions of interest where potential license plates might exist. The common face detection algorithm called Local Binary Patterns (LBP) [23] is used for this purpose. An image may contain one or more regions of interest, each of which goes though the next phases.
- Monochromatic conversion: This part of the sequence is associated with the conversion of the regions of interest detected in the previous phase into black and white images based on the algorithms proposed by Wolf-Jolion [24] and Sauvola [25].
- Character analysis: Once converted to black and white, character analysis algorithms take over trying to find character-sized regions in ascending order of sizes, by starting to look for smaller ones first. Regions with connected blobs that are roughly similar to license plate characters and equal vertical alignment are marked for processing.
- Edge detection: Hough transformation [26] is employed to detect all four edges of the plates since they are linear in nature. This step also takes into account information like character height from the previous phase and the ratio of actual plate width and height depending on the geographical region to make a best guess of the precise position of the plate in the image.
- De-skewing:Now, any rotation or skew that might exist is corrected by remapping the plate region.
- Segmentation of characters: This phase is related to cleaning of the plate region by removing speckles and edges so that they are not mistaken for characters like the letter “l” or the number“1”. Furthermore, all characters are separated, and the vertical histogram is used to detect gaps in characters.
- OCR: This involves analysis of individual characters and the computation of confidence.
- Result generation: In the last part of the sequence, the best possible character combination is searched in the context of the known plate pattern that corresponds to the different regions of the world.

### 3.3. Selection of the Relevant Plate

We know more than one plate may appear in images captured by devices running our application if we have vehicles coming from the opposite direction or vehicles parked, along with the one traveling ahead. Thus, to eliminate the probability of false alarms, we have devised the *same direction test*. Using this test, we can detect vehicles in motion and traveling in the same direction as the user of the application. This way, we can ignore vehicles that are parked or moving in the opposite direction and focus on just the plate of the vehicle in front of us that is moving in the same direction.

**Figure 3:**
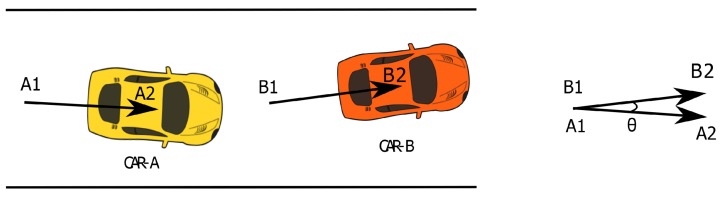
The *same direction test*.

Figure 3 depicts the idea behind the *same direction test*. It involves the construction of displacement vectors for each neighboring vehicle, from the current and previous location information contained in the messages received from them. This information is looked up using the plate number recognized with the help of the smartphone camera and using the plate number as the key for requesting a query to the database storing the messages received. Remember that each vehicle while broadcasting their location information also sends its actual plate number as the message Id. Now, the displacement vector of the neighboring vehicle is compared with the displacement vector of the receiving or current vehicle, and the angle between them is measured. In this figure, CAR-A is following CAR-B, their displacement vectors are constructed from previous locations A1 and B1, and current locations A2 and B2, respectively. If the measured angle (θ) between the displacement vectors is less than a predefined threshold, we consider the two vehicles to be mobile and traveling in the same direction.

Note that only the vehicles ahead will be able to satisfy the *same direction test* when the application is used in scenarios consisting of bidirectional two-lane roads, where one lane is used by vehicles moving in each direction. Thus, this test when coupled with an algorithm that selects the largest recognized plate among all the plates of vehicles moving in the same direction, we are able to successfully select the relevant license plate belonging to the vehicle traveling just ahead.

### 3.4. Distance Calculation

For the calculation of the distance between two vehicles, we rely on image processing and not on GPS data due to their inaccuracy. This is calculated in the following manner.

**Figure 4:**
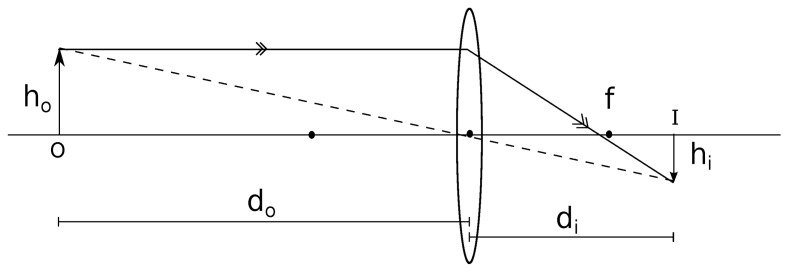
Refraction by convex lens when the object is beyond 2f.

The general equation of the lens is:

(1) $$\frac{1}{f}=\frac{1}{d_{0}}+\frac{1}{d_{i}}$$

where *f* is the focal length of the lens, $d_{o}$ is the distance of the object from the lens and $d_{i}$ is the distance between the lens and the image formed, as seen in Figure 4. However, we also know that,

$$\frac{d_{o}}{d_{i}}=\frac{h_{o}}{h_{i}}$$

(2) $$∴d_{i}=\frac{h_{i}d_{o}}{h_{o}}$$

where $h_{o}$ is the actual height of the object (O) and $h_{i}$ is the height of the image (I) formed on the camera sensor.

Now, substituting Equation (2) in (1), we have:

$$\frac{1}{f}=\frac{1}{d_{o}}+\frac{h_{o}}{h_{i}d_{o}}$$

(3) $$∴d_{o}=f\left(1+\frac{h_{o}}{h_{i}}\right)$$

However, since $h_{i}$ is unknown, it must be calculated indirectly as follows. In the image file of height $h_{f}$ produced by the camera, let the height of object in the image file be $h_{if}$. Thus, on the camera sensor of height $h_{s}$, the height of the real image formed is given by the following equation.

(4) $$h_{i}=\frac{h_{if}h_{s}}{h_{f}}$$

Now, substituting Equation (4) in (3), we have:

(5) $$∴d_{o}=f\left(1+\frac{h_{o}h_{f}}{h_{if}h_{s}}\right)$$

Thus, we use Equation (5) for calculating the distance between the two vehicles from images taken from the vehicle following the other.

### 3.5. Alert User and the Car Ahead

Finally, once the distance between the two vehicles has been calculated, we check if the cars are too close, and in that case, we generate a warning and also caution the driver ahead by sending an alert making use of the GRCBox communication. Now, we have to define a safe distance, for which upon non-compliance, alerts would be generated. Many government agencies like the Road Safety Authority (RSA) [27] of Ireland and the Department of Motor Vehicles [28], State of New York, suggest the two-second rule. According to this rule, the minimum safe distance between two vehicles, one following the other, varies according to the velocity of the vehicle behind. It should be at least the distance that the trailing vehicle would cover maintaining the same speed for two seconds. This should provide enough time for the driver of the car behind to react if the car ahead comes to an abrupt stop. The application is intended for deployment in urban scenarios where speed limits usually vary between 40 and 60 kmph in most countries, resulting in safe distance varying within 22–33 m following the two-second rule. Such large distances are never maintained between vehicles in urban traffic. Therefore, this would render the application unproductive, and it could be considered more of a nuisance if it starts warning drivers when the distance between two cars is over 30 m, causing unnecessary distractions. More reasonable, in this case, would be to maintain a distance of one car length between two vehicles; in other words, the car behind should leave a sufficient gap between itself and the vehicle in front, so that another vehicle could fit in that gap. Since most family-sized cars are within 5 m in length, we select this distance as the minimum safe distance in our application, and thus, if the distance between two vehicles is less than 5 m, it starts displaying warnings.

## 4. The Vehicular Network

The proper functionality of our FCW application is dependent on the availability of a vehicular network for data exchange. However, vehicles used on a daily basis still lack the capability to communicate with one another. Thus, for creating a network of vehicles, we employed GRCBoxes. A GRCBox is a low-cost connectivity device based on the Raspberry Pi [29], which enables V2X communication and encourages the integration of smartphones into vehicular networks. The necessity for a device such as the GRCBox arose due to the difficulty in creating an ad-hoc network merely using smartphones.

**Figure 5:**
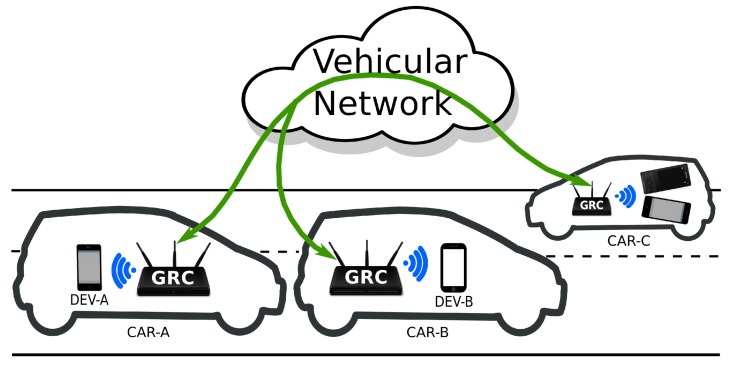
The experiments with our application in a real scenario.

Figure 5 explains how our application works paired with the GRCBox. As can be seen from the figure, each vehicle carries a GRCBox mounted inside to create the vehicular network. Devices of passengers inside the car are connected to the GRCBox, which allows our FCW application to perform data exchange. Here, let us assume that CAR-A and CAR-B are using the designed application, CAR-A approaches CAR-B and fails to maintain the safe distance. Our FCW application detects this, generates a warning for the driver in CAR-A and also sends a message to the vehicle in front taking advantage of the vehicular network created using the GRCBox. CAR-B receives this information from CAR-A; hence, the driver of CAR-B is also alerted.

During the real experiments with our application, cars used in the test had a GRCBox mounted within to be able to communicate. The GRCBox consists of a controller, which is a RaspberryPi, an optional battery as a power source and a USB hub to connect various network interfaces. Each GRCBox has a minimum of two WiFi network interfaces, of which one works as an access point, and the other one is used to create an ad-hoc network. The smartphones and mobile devices used to run our application connect to the GRCBox using the interface working as the access point, and then, the data to be sent are forwarded to the other nodes using the ad-hoc network. Thus, the GRCBox acts as a router for the exchange of data. Even though the GRCBox is supposed to be equipped with 802.11p for vehicular communication, we used 802.11a devices instead, since 802.11p-enabled hardware was not available while setting up the GRCBox to perform the tests. In future experiments, we intend to use 802.11p-compatible hardware to take advantage of the Wireless Access in Vehicular Environments (WAVE) standard.

A minimalist hardware setup of the GRCBox device would require a RaspberryPi as the controller and two USB WiFi interfaces. The current model available officially is the Raspberry Pi 3 Model B+, which is on sale for $35. A USB WiFi interface would cost around $10 each. Thus, setting up a GRCBox device that allows communication among vehicles would cost a total of around $55, which we consider as a very accessible price. The required software that the GRCBox uses can be downloaded, and it comes with a well-documented manual for easy setup. Once the GRCBox device is up and running, our safety application would be able to function taking advantage of the communication networks that the GRCBox supports.

## 5. Results

We have performed various tests both in scenarios with and without mobility. The experiments in scenarios without mobility were to first tune some application parameters and later check the usability of the application in actual scenarios involving mobility.

### 5.1. Static Experiments

In the first scenario, we studied the application performance when no mobility was involved. Photographs of cars that were not mobile were acquired from a parking area, and these images were processed by the Android devices to fine-tune our application. However, before that, we wanted to make sure that our methodology, used to calculate the distance between the cars from the plate size, was able to provide enough accuracy. Thus, we took photographs of a car at a known distance away from the Android camera and processed them to calculate the distance.

Table 1 lists the results obtained, and it can be noted that, as distance increases, the error in the calculated distance also increases, but we are more or less able to get the distance from the images, thus confirming that the developed theory holds. The error involved is nearly insignificant when compared to GPS-related errors. This error could be the result of inaccuracy in the measured plate size in the images; or due to the imprecision in focal length and camera sensor-related information supplied by the Android Operating System (OS), used to estimate the distance between vehicles.

Processing time of images is an important parameter for determining the usability of our FCW application. Thus, in our initial experiment, we wanted to study the time taken by different Android devices to process and identify the license plate for various resolutions. In this experiment, we studied the time taken by five different devices, namely the Nexus 7 tablet, Motorola Moto G-3, Nexus 5X, Nexus 6 and Samsung Galaxy Note 10.1. The Nexus 7 had a quad-core 1.2-GHz processor and 1 GB Random Access Memory (RAM). Similarly, the Moto G-3 possessed a 1.4-GHz quad-core processor and 2 GB RAM. The Nexus 5X was equipped with a 2-GB RAM, a hexa-core processor with four cores running at 1.4 GHz and the other two at 1.8 GHz. The Nexus 6 had a specification of 3 GB RAM and 2.7-GHz quad-core processor. Finally, Note 10.1 came with a 3-GB RAM and 2.3-GHz quad-core processor.

**Figure 6:**
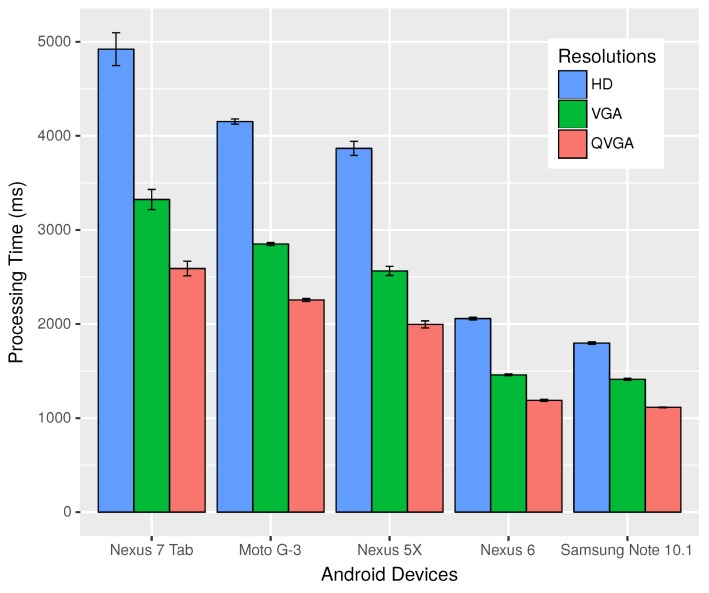
Time taken to process images of different resolutions by different devices.

Figure 6 shows the time taken to process and identify plates in images of resolutions of High Definition (HD), Video Graphics Array (VGA) and Quarter Video Graphics Array (QVGA) for the different devices. The time taken for processing HD images varied from 1.8–4.2 s depending on the device, while it ranged from 1.4–3.3 s when considering VGA, and for QVGA, it was between 1.1 and 2.6 s. Thus, lower resolution images were processed faster, and devices with faster processors performed better. The Samsung Note shows the best performance, followed by Nexus 6, Nexus 5X, Moto G-3 and the Nexus 7 tablet. Note that even with the best processing times achieved by the Samsung Note of 1.8 s for HD, 1.4 s for VGA and 1.1 s for QVGA resolutions, it is still very high when compared with dedicated image processing devices.

The Android OS, while encoding Joint Photographic Experts Group (JPEG) images, accepts a parameter called quality, the value of which can range between zero and 100. The value of zero produces images of maximum compression, while 100 compresses for the max quality. Next, we want to check if this parameter had a role to play in the processing time of images for plate recognition.

**Figure 7:**
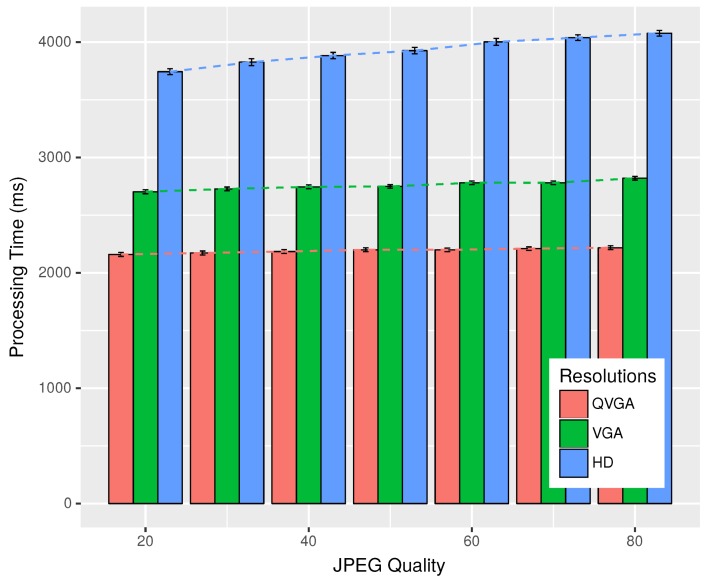
Plate processing time with Moto-G3 for different JPEG qualities.

In Figure 7, we can see that, when the value of the quality parameter was varied from 20–80, the processing time increased sightly for higher values of quality. Higher resolution images were more affected than the lower resolution ones. We have varied the value of the quality parameter from 20–80, as values below 20 resulted in images with very low visually-perceived quality, while values over 80 did not produce any significant improvement. All the processing was done by one device, which was the Moto G-3 in this case. Note that, for the QVGA resolution, the processing time was nearly fixed at 2.2 s, even with the variation of the JPEG quality, while for VGA, it rose from 2.7–2.8 s, and for HD images, it ranged from 3.7–4 s.

So far, we have seen that lower resolution images are processed faster, and the quality of the image has very little or no effect on the processing time for lower resolution images, while in the case of higher resolution, there is an increase in the time taken to detect plates. Another important factor that should also be taken into account is the accuracy or the degree to which the identified plate number and the actual license registration number match. Furthermore, we want to determine whether parameters like image resolution and quality had any effect on the accuracy of the identified plate.

**Figure 8:**
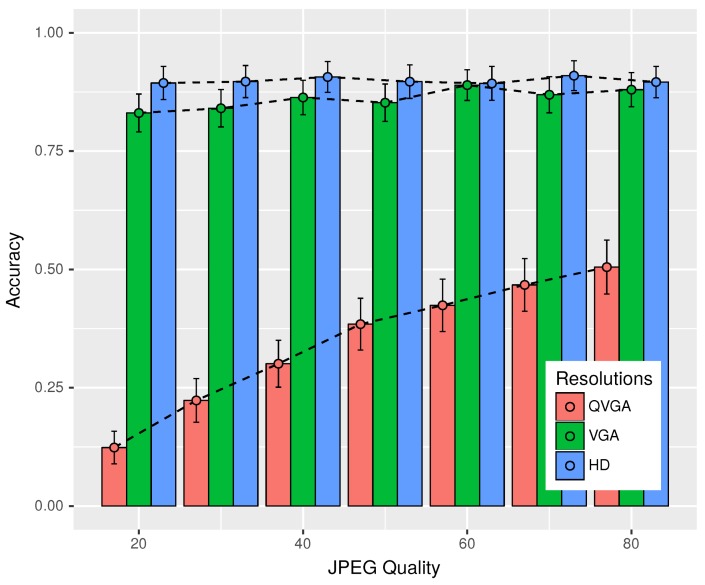
Accuracy of plate recognition for different JPEG qualities.

Figure 8 depicts the effects of JPEG image quality on the accuracy of plate recognition. An accuracy of one reflects an exact match where the identified plate perfectly coincides with the actual plate, while zero indicates no match or no plate was detected in the image, whereas values in between zero and one imply a partial match. Note that the resolution of QVGA showed a huge improvement of accuracy, varying from 0.12–0.5, with a boost in the image quality. For higher resolutions of VGA and HD, it ranged from 0.83–0.89 and lied between 0.89 and 0.91, respectively, depending on the quality parameter. This suggests the use of higher resolutions rather than lower ones for the purpose of our application.

Another physical factor that could effect the performance of our application is ambient light. Thus, to study the effect of lighting conditions, we decided to perform three sets of experiments in variable lighting conditions in the daytime, at dawn/dusk and during the night.

**Figure 9:**
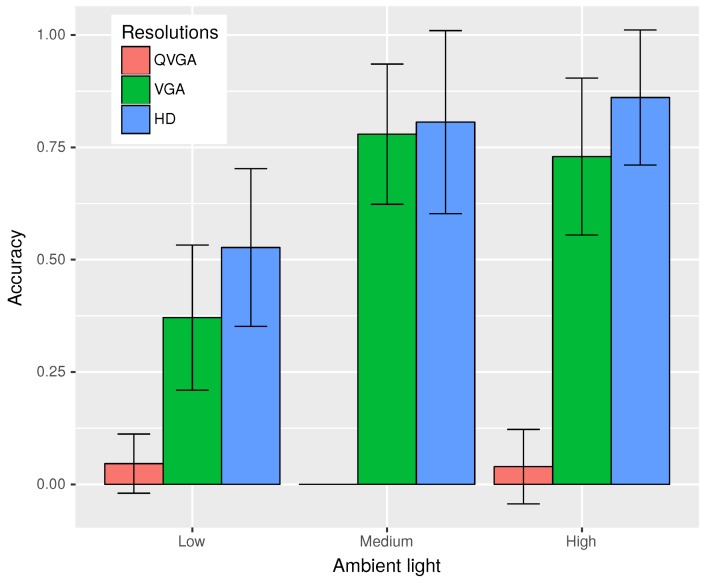
Accuracy of plate recognition in different lighting conditions.

Figure 9 illustrates how the accuracy of the plate recognition changed with altered lighting conditions for various resolutions. Here, low lighting conditions refer to conditions during the night, with the presence of insufficient light from street lamps to nearly no light at all in some images, resulting in grainy, unclear and dark images. While medium ambient light refers to the time when the Sun was just about to rise, or right after it had set. Lastly, high ambient light encompassed all tests that we have performed during the daytime. From the graph, we can see that for QVGA resolution in this set of experiments, we had very little success recognizing plates, even during the daytime; while for VGA images, accuracy varied from 0.37–0.78, with the best performance in medium lighting conditions, as reflections from the plates caused problems in recognition when exposed to high ambient light. For the highest resolution of HD, we were able to achieve accuracy values between 0.52 and 0.86 depending on the amount of ambient light. Note that due to the small size of the dataset used for this experiment, the confidence interval was high in all cases.

Thus, taking into account all that we have learned so far from our experiments, we decided to use the HD resolution due to its dominant performance in different lighting conditions and better accuracy of recognition compared to the lower resolutions. On the subject of regarding which quality settings to use for the HD resolution, we decided on the quality value of 70. Even though, in our experiments in static scenarios, we found out that the quality of images had little effect on the gained accuracy in the case of HD resolution and also resulted in the increase of processing time on using higher quality images. However, taking into account that real scenarios would involve motion, blurring and the effects of vibrations, which would make it harder to recognize the plates, we considered that image quality could have a role to play. Furthermore, from Figure 7 and Figure 8, we had the best accuracy (0.91 or 91%) for HD for a quality value of 70, but of course, the processing time with Moto G-3 was four seconds on average. This is about 8% more than the time required to process HD images of the lowest considered quality settings of 20. Thus, the default settings used JPEG images of HD resolution, with a quality value of 70, which the user can change according to his or her needs.

### 5.2. Dynamic Experiments

In this section, we present the results we have achieved in tests we performed with our Android FCW application, using the settings we have defined from the study of the application in scenarios without mobility. Experiments performed in this section were undertaken using two real vehicles, one following the other at all times, each equipped with a GRCBox for communication, and an Android tablet running the developed application, as shown in Figure 1. The aim of our outdoor experiments with mobility was to try and find a good threshold value for the *same direction test* that was used by the application to discard plates of vehicles not in motion, or coming from the opposite direction, and to see how our application performed in challenging real scenarios.

**Figure 10:**
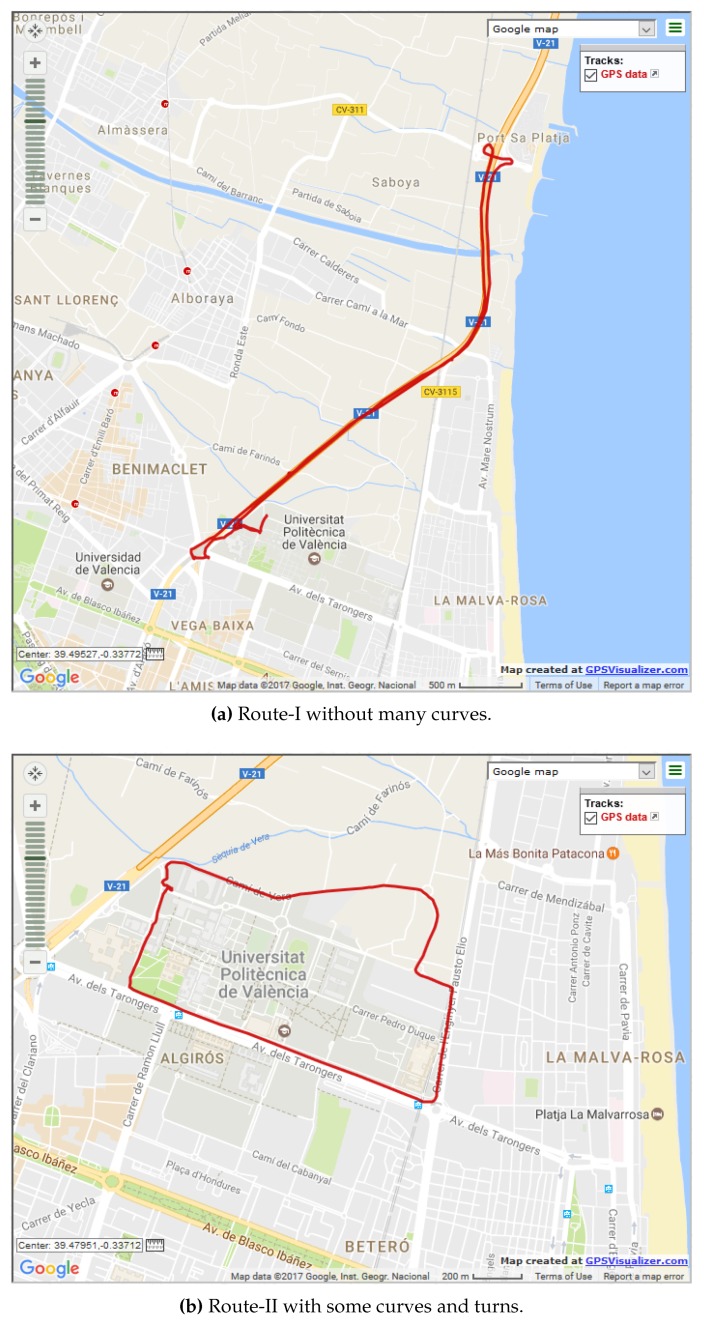
Routes used during the outdoor evaluation.

Figure 10a shows one of the two routes taken during our outdoor experiments. This route was about 9.25 km long with very little turns and curves, hence the vehicles could move at a high velocity. On the other hand, Figure 10b depicts a 3.76-km route, around the Universidad Politécnica de Valencia, where some turns or curves are present and this allowed vehicles to move at a moderate speed.

**Figure 11:**
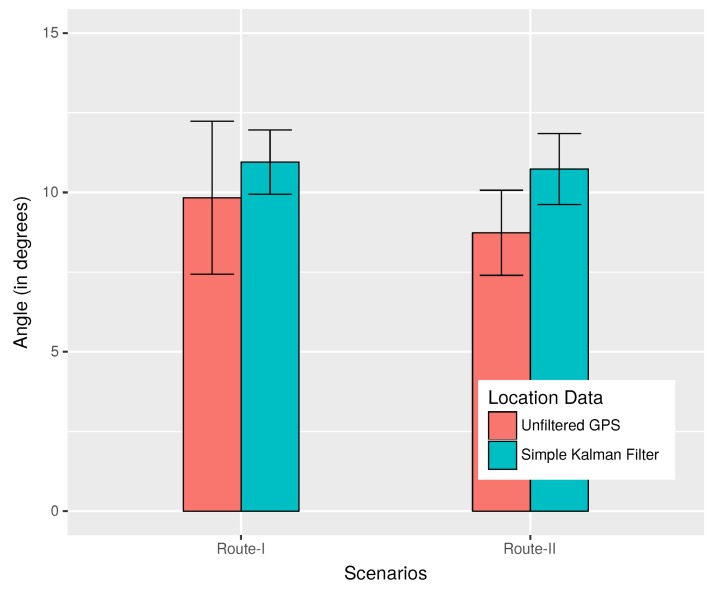
Results of the *same direction test*.

Figure 11 presents the observations from the *same direction test* that was used to detect if vehicles were traveling in the same direction. A comparison has been made between the use of unfiltered GPS locations and Kalman-filtered [30] location data for the evaluation of the *same direction test*. The Kalman filter used here was a simple one that just took into account the location data. From this graph, we can see that the average angles evaluated by the *same direction test* using unfiltered data for scenarios presented in Figure 10a,b were 9.83 and 8.73 degrees; while, using the Kalman filter, similar values of 10.95 and 10.73 were observed, respectively. Since for both filtered and unfiltered data, it can be observed that the worst case values were within 12.5 degrees, 12.5 degrees was selected as the default threshold for the *same direction test* in our application.

**Figure 12:**
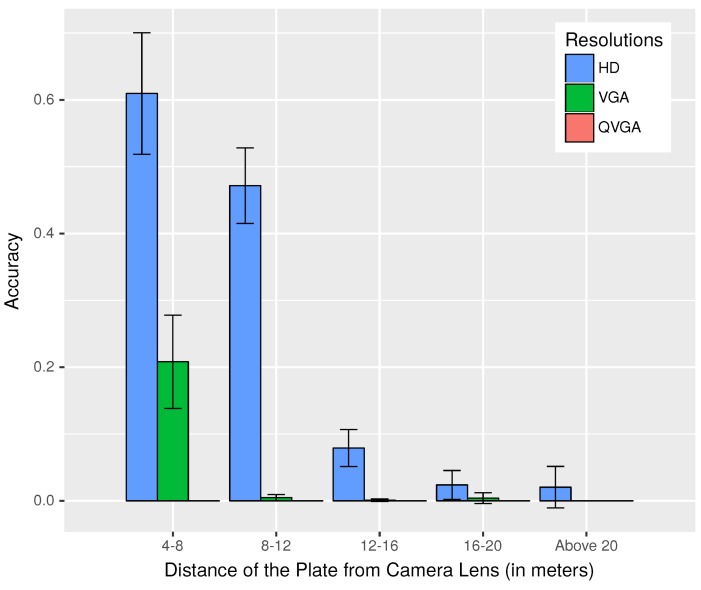
Accuracy of the recognized plates for varying distances during daytime, in scenarios involving motion.

During our assessment of the *same direction test* involving real cars, we also studied the effect of distance on plate recognition as we tried to identify the license plate of the car ahead. Figure 12 displays the results obtained. This graph reassures us of our choice of HD resolution as the default image resolution for our application, as other lower resolutions failed to perform well in this scenario. Let us look closely at the first two groups of observation taken between 4–8 m and 8–12 m, when the vehicle behind moves closer to the vehicle ahead; it is seen that the accuracy of the plate recognition increased. In between 4 and 8 m, the accuracy was about 0.61 for HD images, which was much lower than what we observed in our experiments without mobility. The lower accuracy was the result of an increased number of failures in an attempt to recognize the plate as a consequence of the problems that the device faced to stabilize the images from motion-related vibration issues. Note that, in our experiments, we have also included results when two vehicles were four meters apart, even though warning generation started at 5 m, because the application needed to keep alerting the drivers even when the distance was less than 5 m. We have no results from below four meters, as it was difficult to emulate such dangerous situations in real experiments, and also due to the fact that the plate of the car ahead was outside the captured frame in some of the cases when the two cars were very close to each other.

Finally, we also tried to repeat the same experiments involving motion, during the night time, to make a comparison of how the application performed under low light conditions. We were unable to identify plates during the night using all three different resolutions due to several reasons: the camera equipped in the smartphones found it hard to focus in low light conditions; vehicles usually had lights near the license plate that were present to help illuminate the plate in darkness; however, these lights in our case made it more difficult for the camera to focus, and when the car behind closed in on the car ahead, reflections due to its headlights made the plate illegible. Thus, we see that external factors like ambient light played a huge role and affected the performance of our application. Along the same lines, other environmental factors like rain or fog could have an adverse effect on the application performance as identifying the license plate from images captured under such circumstances would be more difficult.

## 6. Conclusions

We have presented an Android FCW application that uses plate recognition and inter-vehicular communication to alert drivers on getting too close to the vehicle in front. The designed application warns drivers of the vehicle behind and the vehicle ahead, when a defined safe distance of 5 m is not maintained. We have performed various experiments with our application in static scenarios, as well as scenarios involving motion. It was found that the designed application works and achieves better performance with mobile devices with faster processors. From our experiments with the application in static scenarios, we established the default settings used by our application, which includes capturing HD images encoded to JPEG with a quality value of 70. These settings can be modified by the users as per their needs. Our application is able to function effectively in bidirectional two-lane roads, capable of detecting plates of cars coming from the opposite direction or static parked cars and discarding these cases without warning generation with the help of a test that we have designed, named the *same direction test*. This test performs well and helps to make sure that for warning generation, only the distance to the license plate of the vehicle just ahead is considered. The *same direction test* depends on a threshold, and from our experiments with the application involving actual moving vehicles, we established that a threshold value of 12.5 degrees is adequate for this test. The purpose of developing a FCW application for mobile devices was to study the integration of smartphones with VNs for designing cost-effective ITS solutions that can achieve rapid acceptance among the general public. Observations with our FCW application show that integration of smartphones with VNs indeed opens a new horizon for ITS applications. Even though this preliminary version of the application is functional, the mobile devices took too long to recognize license plates (in the order of seconds), which is the biggest reason that might impede the adoption of the solution. Critical safety applications, such as the one we have presented here, need to process more than one image per second, for performance-related reliability. Thus, at the moment, we are concentrated on reducing the image processing time required by the application. Another issue that affected the application was the poor camera quality of the devices used in the experiments. The cameras on the devices were not powerful enough to stabilize images captured when in motion and in conditions involving low light. We are optimistic that the quality of the hardware would only improve with time, and thus, more powerful devices of the future will allow us to take better advantage of this kind of application. Furthermore, a possible improvement for the subsequent version of the application would be making it capable of functioning on roads with multiple lanes for each direction of traffic. In such scenarios, situations might arise where unwanted warnings are generated upon identification of the plate of a vehicle within the communication range, traveling in a different lane, but moving in the same direction as the vehicle behind. This issue has been left as future work and needs to be addressed.

## Figures and Tables

**Table 1:** Comparison of the actual distance with calculations using Equation (5).

Actual Distance (m)	Calculated Distance (m)
3	3.0
5	4.9
8	7.8
10	9.7

## Abbreviations

The following abbreviations are used in this manuscript:

FCW	Forward Collision Warning
GPS	Global Positioning System
HD	High Definition
IEEE	Institute of Electrical and Electronics Engineers
IP	Internet Protocol
ITS	Intelligent Transportation System
JPEG	Joint Photographic Experts Group
LBP	Local Binary Patterns
LiDAR	Light Detection and Ranging
OCR	Optical Character Recognition
OS	Operating System
QVGA	Quarter Video Graphics Array
RAM	Random Access Memory
RSA	Road Safety Authority
V2V	Vehicle to Vehicle
VGA	Video Graphics Array
VNs	Vehicular Networks
WAVE	Wireless Access in Vehicular Environments
